# The Rehabilitation Potential of Neurostimulation for Mild Traumatic Brain Injury in Animal and Human Studies

**DOI:** 10.3390/brainsci13101402

**Published:** 2023-09-30

**Authors:** M. Windy McNerney, Gene G. Gurkoff, Charlotte Beard, Marian E. Berryhill

**Affiliations:** 1 Mental Illness Research Education and Clinical Center (MIRECC), Veterans Affairs Palo Alto Health Care System, Palo Alto, CA 94304, USA; windymc@stanford.edu (M.W.M.); charlottebeard240@gmail.com (C.B.); 2 Department of Psychiatry and Behavioral Sciences, Stanford University School of Medicine, Stanford, CA 94305, USA; 3 Department of Neurological Surgery, and Center for Neuroscience, University of California, Davis, Sacramento, CA 95817, USA; gggurkoff@ucdavis.edu; 4 Department of Veterans Affairs, VA Northern California Health Care System, Martinez, CA 94553, USA; 5 Program in Neuroscience and Behavioral Biology, Emory University, Atlanta, GA 30322, USA; 6 Programs in Cognitive and Brain Sciences, and Integrative Neuroscience, Department of Psychology, University of Nevada, Reno, NV 89557, USA

**Keywords:** traumatic brain injury, rTMS, neuromodulation, rodent model, cognitive neuroscience, executive function

## Abstract

Neurostimulation carries high therapeutic potential, accompanied by an excellent safety profile. In this review, we argue that an arena in which these tools could provide breakthrough benefits is traumatic brain injury (TBI). TBI is a major health problem worldwide, with the majority of cases identified as mild TBI (mTBI). MTBI is of concern because it is a modifiable risk factor for dementia. A major challenge in studying mTBI is its inherent *heterogeneity* across a large feature space (e.g., etiology, age of injury, sex, treatment, initial health status, etc.). Parallel lines of research in human and rodent mTBI can be collated to take advantage of the full suite of neuroscience tools, from neuroimaging (electroencephalography: EEG; functional magnetic resonance imaging: fMRI; diffusion tensor imaging: DTI) to biochemical assays. Despite these attractive components and the need for effective treatments, there are at least two major challenges to implementation. First, there is insufficient understanding of how neurostimulation alters neural mechanisms. Second, there is insufficient understanding of how mTBI alters neural function. The goal of this review is to assemble interrelated but disparate areas of research to identify important gaps in knowledge impeding the implementation of neurostimulation.

## 1. Introduction

This is a narrative literature review of the current research status regarding rodent and human repetitive transcranial magnetic stimulation (rTMS) and other stimulation techniques in the context of *cognitive rehabilitation* from traumatic brain injury (TBI). The goal is to synthesize across interrelated but separate literatures to clarify which clinically relevant translational goals are approaching implementation and to highlight gaps in progress. This review focuses on research involving rTMS and related neuromodulation techniques, including transcranial direct current stimulation (tDCS), transcranial alternating current stimulation (tACS), and deep brain stimulation (DBS), because these techniques exhibit treatment potential for cognitive deficits associated with mild TBI (mTBI) and there are sizeable literatures describing these effects. Thus, this review does not cover psychiatric effects or motor effects, it does not extend to moderate or severe TBI or epileptic patients, and it does not include all forms of neurostimulation.

It is important to reduce the silos between human and rodent researchers. Rodent models inform neurobiological mechanisms, which are essential for any intervention. Rodents’ accelerated lifespans and reproduction rates permit testing treatments in hundreds of mice per year. In turn, this streamlines and accelerates clinical trials in humans. The research will be further advanced by a systematic hypothesis-driven approach to experimental design regarding stimulation parameters. This review emphasizes the difficulty in synthesis and comparisons between protocols when there is little consistency in paradigms [1]. This issue can be remediated by integration across fields and with emphasis on mechanisms—even in the face of a heterogeneous condition like mTBI. This discussion will hopefully accelerate progress in addressing mTBI via collaborations between researchers studying different models who are incorporating neurostimulation and neuromodulation approaches.

## 2. Mild TBI (mTBI) and Repeat mTBI (rmTBI) Are Serious Public Health Problems

The question of when, where, and how neurostimulation can treat TBI recovery arises from the daunting prevalence of TBI. Worldwide there are an estimated 69 *million* TBIs each year. Following *severe* TBI, outcomes are variable, with mortality rates ranging from 24% to 35% [2]. Survivors experience a range of outcomes from permanent vegetative states or severe disability on the low end to good recovery on the high end. Critically, outcomes depended on factors including injury type (i.e., subdural hematoma and diffuse axonal injury), age at injury, duration of hospital stay, presence of persistent coma, or high intracranial pressures) [2,3,4]. Depending on the clinical population, the proportion of favorable outcomes (Glasgow Outcome Extended >4) ranged from 21 to 74%. However, the vast majority of TBIs (>80%) are classified as *mild* in both civilian [5] and active duty populations [6]. MTBI is not typically associated with gross anatomical damage, and the majority of patients return to work well within a year (GOS-E > 5; [7]). Behavioral symptoms of mTBI typically resolve over days to months [8] and without evidence of permanent damage [9]. However, an estimated 5–15% of mTBI cases exhibit enduring cognitive deficits even *years* post-injury [10,11]. Patterns of deficit are idiosyncratic because injuries are heterogeneous [12]. Notably, this is separate from those individuals whose *physical* symptoms persist, a condition termed *post-concussive syndrome,* which has its own attendant literature for diagnosis and treatment (reviewed in [13,14,15,16,17]). Critically, rates of TBI are rising worldwide, thereby increasing the international burden of TBI [18].

In the United States, some 5.5% or 40,000 emergency department visits for TBI evaluation involved individuals reporting a rmTBI within 1 year [19]. Repeat mTBI (rmTBI) is associated with worse long-term physical and cognitive outcomes [20] and is a key antecedent to chronic traumatic encephalopathy (CTE; [21]. Furthermore, annually, an estimated *3,000,000* individuals worldwide experience rmTBI. These mTBI and rmTBI patients are seldom treated for more than a few months. Importantly, it is now acknowledged that a mid-life mTBI is a modifiable risk factor for dementia [22,23]. Unfortunately, there is little that can be done to treat mTBI apart from addressing physical symptoms, prescribing moderate rest, and slow return to activities; in more severe cases of TBI, there can be longer-term rehabilitation provided by allied health fields [24]. There are significant gaps in the understanding of long-term physical, behavioral, cognitive, mental health, and health cost implications attributable to mTBI, which hampers progress in treatment development. Uncovering these mechanisms and harnessing the potential for neuromodulation may allow us to treat those patients with long-term disability and, perhaps, reduce recovery times for those patients who otherwise take months to return to their pre-injury cognitive state.

## 3. Example Given: Persistent Cognitive Deficits

A small literature characterizes lasting effects even after *concussion*, which is an mTBI that has no associated findings from neuroimaging (e.g., computed tomography: CT or magnetic resonance imaging: MRI). One cognitive domain revealing deficits is working memory (WM), the ability to hold a small amount of information over a brief period of time. For example, remembering the name of a new acquaintance to introduce them to another person. This may be because WM engages large swaths of the brain to encode, maintain/manipulate, and retrieve information. Immediately following pediatric and adolescent rmTBI, it is clear that cognitive processing slows [25] and WM performance falls [26,27,28,29]. Perhaps surprisingly, recent findings from the Transforming Research and Clinical Knowledge (TRACK-TBI) longitudinal study reveal that 53% of mTBI patients bear physical or cognitive traces for at least one year post-injury [30]. Psychiatric and behavioral health are also affected. Data from the Adolescent Brain Cognitive Development Study reveal that lasting emotional (anxiety) and behavioral (aggression, social, thought, conduct disorders) significantly increase for *girls* with rmTBI, whereas a past mTBI increases anxiety and attention deficits [31]. In contrast, for *boys*, a history of mTBI (hmTBI) or rmTBI heightened aggression [31]. Yet, little research is devoted to disparate health outcomes after mTBI or rmTBI across the lifespan.

Because executive function, including WM, is essential for successful academic performance, it is surprising that *undergraduates* with a hmTBI (mean ~4 *years* post-injury) or rmTBI exhibit WM impairment at the group level. Performance deficits emerge in a WM change detection task requiring retention of three-color patches for 500 msec [32]. These WM deficits extend across WM tasks, stimuli, and retrieval demands [32,33,34]. Other deficits in hmTBI include impaired performance in attentional tasks such as multiple object tracking [35] and visual cueing [36]. Looking at the heterogeneity in the hmTBI population indicates that group effects are driven by ~30% of hmTBI participants who perform >2 standard deviations from the control mean. Inherent to mTBI is *heterogeneity* [37], with multiple variables influencing long-term outcomes, including pre-TBI health and socioeconomic status [38]. Indeed, most people fully recover, but not everyone. It is important to identify the characteristics that are most predictive of *poor* recovery trajectories.

## 4. Linking mTBI Sequelae to Dementia Risk

As noted, rehabilitation is important because TBI is associated with an increased risk of dementia [23]. HmTBI, especially rmTBI or moderate/severe TBI, increases the likelihood of dementia [39,40]. Older Veterans with hmTBI, regardless of when experienced, also show increased dementia risk [41]. Recent findings link acute and subacute mTBI recovery with another risk factor, the apolipoprotein epsilon 4 (APOE e4) genetic polymorphism. These data revealed that APOE e4 carriers with mTBI had even lower cerebral oxygen saturation [42] and irregular neural slow wave activity [43] and lower perfusion [44] than non-carriers. In short, in mTBI, recovery is complicated by interactions with other risk factors that are only beginning to be understood.

Research at the cellular and molecular levels is characterizing how mTBI increases dementia risk. Acutely, pathological amyloid and hyperphosphorylated tau (pTau) can develop and can persist for years post-injury [45]. Proper clearance of pathological proteins is critical for maintaining brain health and preventing dementia [46,47]. MTBI may also induce microglia to express a pro-inflammatory phenotype, which may impair debris clearance and is seen in neurodegenerative diseases [48]. Thus, microglia function may mediate the progression from TBI to degeneration [49]. Similarly, hyperreactive astrocytes have been reported following injury [50] and in degenerative diseases [51,52].

At the neuronal level, axonal injury from shearing forces and swelling following TBI may contribute to degenerative physiology [53]. White matter atrophy can occur in chronic post-injury pathology [54] and may be related to pTau [55]. Recent reports identify white matter loss as a key contributor to cognitive decline in healthy aging [56] and neurodegeneration [57]. As glial cells support many aspects of neural communication and health, any long-lasting, unhealthy post-injury alterations would reduce neural health and plasticity. Indeed, mTBIs and advancing age reduce the brain’s regenerative capabilities [49,58,59,60].

CTE is a degenerative disease linked to rmTBI, especially in high-contact sports athletes [61]. CTE is characterized by loss of the cognitive control required for WM and attention, and neurobiologically, with cell loss and large accumulations of pTau in neurofibrillary and astrocytic tangles [21,62,63,64]. These changes are likely due to axonal injury and exacerbated by impaired ability to repair damage and clear debris [65], resulting in aggressive neurodegeneration. However, even without CTE, the downstream pathological response to rmTBI is related to other degenerative diseases and could be a contributing factor to future disease development. Researchers must uncover reliable biomarkers to determine if this mechanism can be modulated by neurostimulation [66]. Biochemical research in animal models will improve our overall understanding of this transition and focus the search for predictive biomarkers.

## 5. Rodent Models Elucidate Mechanism and Inform Treatment

On the other side of health science biology, rodents with rmTBI exhibit parallel cognitive deficits akin to WM deficits observed in humans. Models of rmTBI in mice [67,68] and rats [69,70,71] produce cognitive deficits. In rodents, one mTBI is typically not sufficient to impair behavior, but rats with rmTBI demonstrate WM deficits in *novel object recognition* [72,73] and *novel context mismatch* [74], as do humans.

Microglia and inflammation. Rodent models clarify inflammatory signaling and microglia activation states post-TBI, both of which are linked to neuronal loss [75]. In mice, several days after the *control cortical impact* paradigm, microglia activation shifts to pro-inflammatory phenotyping, including the production of superoxides [76]. As mentioned, microglia dysfunction is linked to CTE following injuries, so research in animal models should focus on countering overactivation and subsequent downstream consequences. For example, inhibiting the production of superoxides reduced oxidative damage [76]. Similarly, the pro-inflammatory signal *podoplanin* increased in mice following injury, with some in vitro evidence that knocking down podoplanin reduces pro-inflammatory microglia states [77]. As microglia dysfunction is a favored underlying component of Alzheimer’s disease (AD) in rodents and humans, research in rodent models may further inform effective preventative therapeutic strategies.

Sequencing. Techniques such as RNA sequencing can provide a means for understanding the biological components of mTBI and cellular loss. This technique involves transcriptome-wide analysis of gene expression and has provided novel insights into the biochemical milieu of mTBI in addition to other domains of neuroscience. Immediate changes in gene expression following mTBI include many pathways involving cellular death, inflammation, and astrocytosis, whereas long-term alterations include genes related to metabolism and neurodegenerative processes [78]. In rmTBI mice, memory deficits persist 3 months following injury with congruent transcriptomic changes in memory pathways, including long-term potentiation (LTP) [79]. It is difficult to design medications to target all the differentially expressed genes as an intervention, but these approaches expand the number of possible treatment targets. Unfortunately, rodent researchers must be cognizant of differences in metabolism and overall brain structures between species [80] when extrapolating towards human mechanisms. Additionally, most behavioral or biochemical measurements occur at one point in time, typically at the end of the study. However, we can use electroencephalography (EEG) to safely and noninvasively monitor neural activity longitudinally in both rodents and humans. Moreover, unlike pharmacology, neurostimulation has the potential to modulate multiple treatment targets across levels of scale (e.g., metabolism, neurotransmission, inflammation).

## 6. Electroencephalography in Humans and Rodents

Studies using EEG can track disease time course and treatment effects in animal models and can be readily translated into human research. EEG is a method for recording changes in electric fields generated from within the brain with excellent temporal resolution. When there is a separation of charge between two regions, say between cell bodies in a pyramidal layer and their dendrites in the molecular layer, this leads to the generation of a dipole moment and an electric field. Changes in electric fields can be driven by action potentials, local movements of ions down an axon, and also by more local events such as postsynaptic excitatory and inhibitory potentials. As the nervous system is well organized, with layers of pyramidal neurons and collections of synapses, the dipoles generated by thousands of cells sum to generate the electric fields recorded on the EEG. Patterned oscillatory activity captured in the EEG reflects coordinated neuronal activity [81]. Importantly, EEG is practical. EEG systems are affordable and easily deployable. They are safe and can be applied at any time during the course of recovery (e.g., hours to years after injury), used during the performance of cognitive tasks, and, as the temporal resolution is sub-second, neural activity can be attributed to different aspects of learning and recovery.

## 7. Electroencephalography in hmTBI

Any TBI inducing a detectable gross anatomical change or acute sequela is not mild. Thus, imaging (MRI or positron emission tomography PET scans) is appropriate for moderate/severe TBI in animals and humans alike but is generally blind to mTBI [9]. Fortunately, EEG provides a widely available tool sensitive to neural changes after TBI, including in oscillatory activity, event-related potentials (ERPs), connectivity, and other analyses. In EEG, there are multiple frequency bands exhibiting characteristic oscillatory activity. WM involves theta band activity (4–8 Hz human, 4–12 Hz rat) [82,83]. In both species, mTBI suppresses theta power [84,85]. EEG power spectra demonstrate restoration in theta power over the first 6 months of mTBI recovery [86]. Similarly, theta *phase synchrony*, a measure of oscillatory coherence between two electrodes, recovers during several months post-injury in humans [87]. Theta measures fall with mTBI and return slowly over time.

EEG data collected during repetitions of the same stimulus can be averaged to calculate event-related potentials (ERPs). With regard to ERP findings after mTBI, rmTBI ERP amplitudes are lower during WM [88,89,90], semantic processing [91], and memory encoding [92]. However, a key feature that enhances clinical relevance is that ERP amplitude improves over time, providing a measure to track the degree of recovery [89]. EEG connectivity analyses can reveal disconnection post-TBI due to cortical *or* white matter damage. For example, there is abnormal coherence during a WM task but not during rest after mTBI [93]. Indeed, resting state EEG data are sufficiently sensitive to predict the classification of acute mTBI status [94] and hmTBI status in retired professional football players [95]. Pairing EEG with other imaging modalities, such as diffusion tensor imaging (DTI), permits detailing of lasting white matter damage due to TBI and can be evaluated in both animal models as well as clinically. For example, one combined DTI-EEG study in blast-mTBI patients linked the EEG-derived atypical phase synchrony between frontal lobes with altered fractional anisotropy in white matter tracts derived from the DTI [96]. Because EEG is available, affordable, and versatile, it provides an incredibly important tool for researchers and clinicians interested in documenting recovery and describing mechanisms after an mTBI.

A fundamental challenge in studying mTBI in patients is the heterogeneity of injury (i.e., impact site, severity) and the challenge of capturing longitudinal data. For example, it is not always possible to test a patient within the first 3–6 h post-injury. Patients may not reliably return for follow-up evaluations, particularly for research. Rodent models, however, allow for the investigator to model a range of injury types and severities, including, for example, the *controlled cortical impact* injury, which can create a focal injury, the *lateral fluid percussion*, a mixed diffuse/focal injury, and the weight drop and CHIMERA models more diffuse injuries [97]. Each of these injuries can be scaled for severity (e.g., mild, moderate, or severe), and both chronic constriction injury (CCI) and fluid-percussion injury (FPI) can be applied over different regions of the brain. There is also a model of ballistic penetrating injury [97]. Thus, the research community has the ability to model much of the heterogeneity observed in humans, and individual laboratories often choose to model a specific injury type, allowing for higher reproducibility with smaller sample sizes. After injury, rodent researchers may then study the nature of such injury using clinically relevant tools such as EEG, MRI, or PET paired with distinct laboratory techniques such as biochemistry. Rodent models provide the opportunity to identify mechanisms and targets for therapy and novel, innovative strategies for treatment. For example, we make the case that electrophysiology can be used to identify a mechanism related to cognitive dysfunction and a target for treatment. What is critical, however, is for researchers to consider optimal neuromodulation parameters for treatment. These include identifying ideal targets, types of stimulation (e.g., rTMS, DBS), stimulation frequency, and duration of stimulation, amongst others.

## 8. Neurostimulation Techniques (Table 1)

Unlike activating/inactivating a receptor or scavenging reactive oxygen species (ROS), it is unclear that a pharmacological intervention can modulate oscillations in general, let alone in a specific frequency band. Neuromodulation, however, can modulate neural activity in ways that result in changes in theta, delta, and gamma oscillations (reviewed in [98]). There are multiple techniques for stimulating neural activity non-invasively, such as tDCS, tACS, and rTMS, where investigators can place a combination of anode(s) and cathode(s) on the scalp (tDCS, tACS) or use magnetic stimulation to drive neural activity in specific cortical regions (rTMS). These stimulation paradigms are flexible as they can target a variety of cortical regions, can be varied in frequency and amplitude, and, critically, are non-invasive. Noninvasive brain stimulation likely interacts with myriad receptors, neurotransmitter systems, and ion channels, thus creating the potential for a variety of neural mechanisms depending on the task. There is FDA approval for rTMS applied to the left dorsolateral prefrontal cortex as a target for depression [99], and although many researchers chose this region for mTBI, some neuromodulation publications target different sites to address other issues, including verbal retrieval [100] and chronic pain [101]. There are also well-characterized invasive techniques for neuromodulation, including DBS. Although DBS requires a surgical implantation of electrodes and an internal pulse generator, the lead can be placed directly in a target region, including subcortical regions that are typically inaccessible using extracranial stimulation. DBS, like other approaches, has the flexibility to alternate the amplitude and frequency (Hz) of stimulation. However, an added benefit of DBS is that stimulation can be applied *continuously* or intermittently. Each of these stimulation techniques will be considered below for their potential to modulate theta oscillations and outcomes in the context of mTBI.

**Table 1:** Popular methods of neurostimulation in humans and rodent models. The optimal parameters for improving cognitive performance in people with mTBI are still under investigation. To date, the most common target is DLPFC, but there is some variability across other key parameters. Thus, we include references to the biological systems that are targeted to help with the conceptualization of the underlying mechanism for each approach. Up and down arrows refer to increases or decreases due to neurostimulation.

Stim.	Clinical Applications	Method	Limitations	Advantages	Biological System Target
**Repetitive Transcranial Magnetic Stimulation (rTMS)**	- ↑ post-encoding memory (mice) - ↓ depression - FDA-approved for chronic pain, major depressive disorder, and obsessive compulsive disorder	- Applies alternating magnetic fields generated by coil placed on scalp	- Rodents: under anesthesia/restraint - Human: office visit required; coil type dictates depth - Little systematicity across investigations	- Noninvasive - Focal - Minimal side effects - FDA approval for some	- ↓ apoptosis - ↑ neural survival - ↑ cholinergic and neurotrophic factor signaling in mice (important for cognition and TBI recovery) - ↑ metabolism - alters neural excitability
**Transcranial Direct Current Stimulation (tDCS) and Transcranial Alternating Current (tACS)**	- ↑ task performance - ↑ WM - ↑ spatial WM (rats) - ↑ ChAT potentially reduces transition from injury → degeneration	- Electrical current through two scalp electrodes - Current/field modified for depth/intensity	- Cognition benefits are temporary - Specific optimized timeframe - Not focal - Requires frequency-specific tailoring to individual	- Noninvasive - Affordable - Potentially self-administrable - Safe - Can target deeper structures (e.g., hippocampus)	- ↑ theta synchrony, phase synchrony, phase–amplitude coupling, and theta-gamma cross-frequency coupling - ↑ plasticity and BDNF release in the hippocampus and frontal cortex (rodent) - ↑ ChAT in hippocampus (rodent) -Alters resting potential
**Deep Brain Stimulation** **(DBS)**	- Rehabilitate from cognitive and neurological disturbances - May slow cognitive decline in AD model rodents - FDA-approved: PD, OCD, epilepsy, dystonia	- Surgical implantation of embedded electrodes in brain	- Risks associated with brain surgery - Invasive procedure	- Highly Focal - Low maintenance - Independently operating - Can be adapted for closed-loop	- ↑ metabolism

## 9. rTMS and tDCS Benefits in Rodents after TBI

rTMS. A decade of theoretical pieces [102,103,104,105] and review papers [103,106,107,108,109,110] enthuse the potential of rTMS to treat the long-term cognitive and psychiatric consequences of TBI with a more tempered set of outcomes. rTMS applies alternating magnetic fields generated from a coil placed on the scalp to non-invasively induce a neural response with minimal side effects [111]. High-frequency stimulation (>5 Hz) is thought to induce an excitable response and increase cerebral blood flow, whereas low-frequency stimulation (<1 Hz) typically induces inhibitory effects [112]. In clinical trials, researchers vary stimulation intensity in terms of percent motor threshold (MT). MT is determined in individuals by stimulating the hand area of the primary motor cortex and looking for the lowest intensity pulse that elicits muscle twitches half of the time. Although there is little systematic investigation on the effects of stimulation intensity, rodent research has shown that intensity can influence biochemical response [113]. Overall, there is evidence that rTMS is a potential treatment modality to remediate cognition, but more research needs to fully uncover the optimal parameters and interventions to stop degeneration.

Biochemical changes follow rTMS in rodents with TBI, specifically in terms of neural survival and decreased apoptosis, but with mixed effects on behavior [114,115,116]. This may be because the optimal rTMS parameters are undetermined [117] or because most rodent models administer rTMS while under restraint or anesthesia [118]. Newly developed methods demonstrate how to provide consistent rTMS to mice without restraint or anesthesia [119], and this method improved post-encoding memory in mice [120]. Indeed, rTMS in unrestrained triple transgenic Alzheimer’s mice (3×TgAD) mice improved cholinergic and neurotrophic factor signaling, both of which are important for cognition and mTBI recovery [121].

A pediatric rat model of controlled cortical impact TBI applied 4 weeks of 20 Hz rTMS and improved CaMKII signaling two weeks after the stimulation ended [122]. Although this study provides some evidence that rTMS may elicit long-term synaptic plasticity-related benefits, the use of rTMS as a means to prevent degeneration in hmTBI is underexplored. As hippocampal dysfunction is a major source of memory loss after mTBI [123] and degrades early in many degenerative disorders, whether and how neurostimulation could be beneficial is yet to be determined. Although directly targeting the hippocampus in humans with rTMS is nearly impossible, stimulating highly interconnected regions such as the inferior parietal cortex may increase connectivity and memory performance in human participants [124].

tDCS. Another method, transcranial direct current stimulation (tDCS), may more flexibly target structures like the hippocampus and is a promising technique for post-injury rehabilitation. Instead of an electric field via magnetic induction, tDCS sends a small electrical current through the scalp to interface with the brain through two surface electrodes [125,126,127]. Excitatory and inhibitory stimulation may be achieved through positive (anodal) or negative (cathodal) current generation [128,129,130]. A recent meta-analysis recommended tDCS as a means to induce plasticity and brain-derived neurotrophic factor (BDNF) release in the rodent hippocampus and frontal cortex [131]. Along these lines, medial prefrontal *cortical epidural* direct current stimulation improved spatial working memory in healthy rats [132]. As an intervention, tDCS has improved spatial cognition in the acute phase following a lateral fluid percussion TBI [133]. Unfortunately, the benefits on cognition were temporary, and neurotrophic factor signaling improved only when treatment was administered two weeks after injury rather than one week after injury. However, the results are promising in that tDCS can modulate cognition when provided within an optimized timeframe.

Frontal tDCS applied to rats 24 h after a hippocampal injection of amyloid showed improvements in performance on the Morris Water Maze Task with high amplitude (100 uA and 200 uA) stimulation, which was accompanied by an increased expression of choline transferase (ChAT) in the hippocampus [134]. ChAT is an important marker for cholinergic function and is dysregulated early in the course of neurodegenerative diseases in humans. Thus, this may be helpful in reducing a transition from injury to degeneration. However, as the intervention was provided in a short time frame following amyloid injections, it is unclear how tDCS would fare in a scenario with longer-term damage, which is more common in human degenerative pathology. To address this, researchers applied 3 weeks of tDCS to the 3×TgAD genetic mouse model of AD, but no biochemical or behavioral benefits were found [135]. One potential confound is that a much lower amplitude was utilized in this study (50 uA), as more research needs to be conducted to find the correct stimulation patterns for different types of brain diseases. On the other hand, implanting electrodes directly into the brain allows for a more controlled targeting approach, which provides an efficacious strategy in disease states needing more frequent or constant intervention.

DBS. Deep brain stimulation via implanted electrodes is promising in its ability to rehabilitate those suffering from debilitating cognitive and neurological disturbances and has emerged as a highly efficacious treatment for Parkinson’s disease in rodents [136,137] and humans [138]. Although DBS provides a very site-specific interface, the overall excitatory or inhibitory effect may depend on the neural composition within the locus of stimulation [139]. There is some evidence that it may slow cognitive decline in rodent models of AD [140,141]. In addition, DBS in the laboratory setting has been able to improve cognitive performance in rodent models of epilepsy as well as moderate–severe TBI [142,143,144,145,146]. Although there are risks with any surgery [147], DBS is FDA-approved for treating several disorders, including Parkinson’s disease, dystonia, obsessive compulsive disorder, and epilepsy, with others on the horizon. It is important to consider the specific needs of the patient and the cost–benefit profile when considering the utility of invasive as compared to non-invasive approaches for neurostimulation.

**Figure 1:**
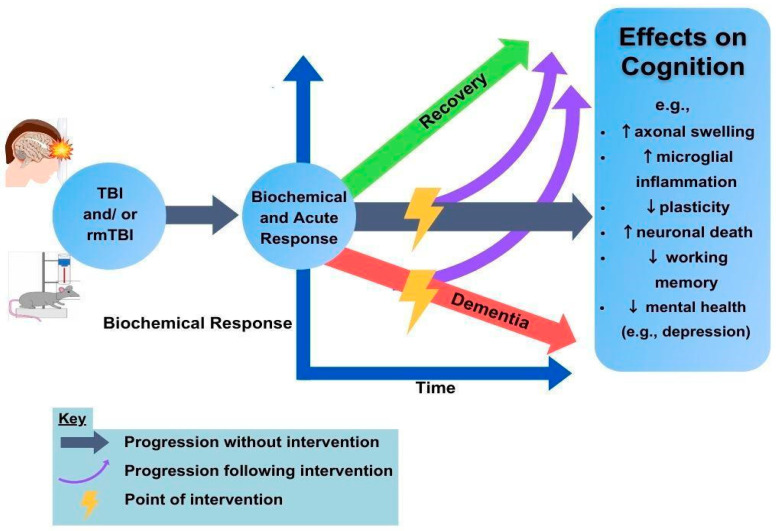
TBI and neurostimulation. The human and rodent TBI populations provide a rich set of data in which to identify targets for rTMS or neuromodulatory recovery (lightning bolts). The ability to understand the unfolding biochemical response over time and the resulting consequences on cognition are aimed at improving outcome trajectories (purple arrows) to benefit recovery. Green and red arrows indicate good and poor long-term outcomes, respectively.

Translatability concerns. Future directions in rodent research are abundant but should prioritize translatability. Rodent research involves precisely phenotyped injuries in genetically identical animals, creating an environment for results with large effect sizes. While animal models are, therefore, critical for fine-tuning a mechanistic understanding, as well as assessing potential safety and efficacy profiles, translation to human rehabilitation remains a challenge [148]. Therefore, researchers should aim towards systematically investigating multiple rodent genotypes and injury models when utilizing brain stimulation for rehabilitation. Questions regarding optimal timing post-injury and treatment parameters are still to be answered. Additionally, it is unclear how much change in cognition is sufficient to merit neurostimulation as an interventional strategy. Continued collaboration between scientists and clinicians will build upon the current knowledge base to tackle these questions, see Figure 1.

## 10. rTMS and tDCS Benefits in Humans after TBI

A major challenge in building synthesis for treatment approaches across noninvasive brain stimulation tools is that the only consistency across labs is *inconsistency*. Studies are often underpowered and variable in terms of parameters, tasks, and populations. In this section, common threads are extracted from the field by focusing on recent reviews and meta-analyses to pinpoint a signal amidst considerable noise. It is important to note that stimulation to improve cognition in TBI patients produces mixed findings. In short, *some* patients under some protocols show some temporary cognitive benefits [126,149,150,151]. Unfortunately, rTMS and tDCS are not panaceas, and further optimization is needed (reviewed in [107]). More specifically, effects are usually limited to an aspect of performance rather than broadly generalizable across cognition. It is important to determine the full range of effects by including multiple tasks. For example, one paper applied dorsolateral rTMS (or tDCS) and improved WM performance in TBI survivors but found no effects on processing speed, verbal learning, verbal fluency, or social cognition [152].

With regard to cognitive improvement with rTMS in TBI, more broadly, there are intriguing findings but little large-scale success and little systematicity across investigations. Several studies modify rTMS by presenting stimuli in theta bursts, using much briefer sessions (>5 min) than the typical rTMS (20–40 min), but having long-lasting benefits primarily in treating depression [153,154,155]. Theta burst stimulation (TBS) is thought to more closely mimic natural neural rhythmic activity and may be excitatory or inhibitory, depending on the pattern [154]. Case studies revealed improved attention in one hemispatial neglect patient after *continuous* theta burst TMS (cTBS), which is associated with induction of long-term depression [156,157], and a different patient with upper motor neuron damage showed improved reaching after three months of rTMS [158]. The application of *intermittent* theta burst stimulation (iTBS) targeting the DLPFC in patients 1 month post-mTBI provided no cognitive benefit [159] but did help control participants [160]. ITBS is associated with the induction of long-term potentiation [153,157]. Thus, further advances in pulse sequencing may be associated with a full palette of mechanisms that can be selected for tailored effects.

Meta-analyses evaluating the effect of FDA-approved rTMS for depression confirm efficacy, e.g., [161,162,163,164], but effects do not appear to extend to provide generalizable cognitive benefits in TBI patients (reviewed in [165]). One paper found the expected relief from depression, but cognitive tests revealed only a moderate benefit to visuospatial memory (Brief Visuospatial Memory Test) and a minimal benefit on selective attention (Stroop color–word task, Trails Making Tests A, B) [140]. The mechanism of relief is attributed to stronger resting state connectivity [162]. Similarly, Veterans with depression (*n* = 321), or depression *and* hmTBI (*n* = 337), showed relief from depression after 30-rTMS sessions [166]. Their interpretation was that hmTBI did not interfere with the benefit of rTMS for depression, but neither did rTMS provide additional benefit. These data provide reassurance that rTMS is safe for use in people with depression and hmTBI, two commonly comorbid conditions in Veterans. A second meta-analysis found that those with TBI showed that rTMS reduced depression and chronic pain but did not improve cognition [167]. To date, evidence is lacking that rTMS using the FDA-approved depression protocol provides any benefit to cognitive performance (reviewed in [165]).

Such inconsistent benefits of rTMS in TBI may reflect the push to rapidly develop and deploy technologies without understanding the underlying mechanism(s) of cognitive impairment. Better targets, for example, strengthening theta oscillations post-TBI, may produce better outcomes, or some other putative mechanism may be superior. However, it is also possible that there is no optimal target for theta rTMS. In summary, to effectively translate neuromodulation modalities to rehabilitate TBI, it is critical to move from inconsistency to interdisciplinary synthesis and hypothesis testing. Next, we explore such a combined effort to take a deeper dive into theta oscillations, which are at the forefront of TBI research and rehabilitation efforts.

## 11. The Role of Theta Oscillations in Plasticity and Learning

The question is clear: In the context of TBI-induced cognitive deficits, what is a reasonable therapeutic target? One potential target is slow wave theta oscillation. Theta oscillations are attractive because they are prominent across the brain. Theta oscillations are present in hippocampal local field potentials (LFP) [168], and they modulate hippocampal LTP [169,170], which underlies learning and memory [171,172]. LTP is more robust when high-frequency stimulation coincides with the theta peak [173,174,175], and LTP is diminished if theta is attenuated [176]. Indeed, theta oscillations coordinate local and distal neural networks. For example, there are coherent theta oscillations between the hippocampus and prefrontal cortex [177,178] and cross-frequency interactions such as *phase–amplitude coupling* between theta and gamma. Specifically, as theta power increases, so does in-phase gamma power in both rodents [179,180,181] and humans [182,183,184]. Disrupted coupling worsens cognitive performance [185,186]; reviewed in [187,188].

Coherent rhythms are also critical for spatial navigation as hippocampal place cell activity is coupled to oscillatory activity, termed *phase precession* [189,190]. Specifically, as animals enter a hippocampal place field, action potentials initially occur late in the oscillation, but as the animals approach an edge, action potentials progressively occur earlier [189,190,191]. Critically, similar to rodents, in humans, intracranial recordings of local field potentials from epileptic patients revealed that hippocampal theta oscillations are prevalent during spatial navigation (virtual and real-world) [192,193,194,195], successful object recognition tasks [196,197,198,199], and successful recall [200]. In summary, theta oscillations play a prominent role in coordinating neural activity associated with learning and memory.

## 12. TBI Disrupts Theta Oscillations in Humans and Rodents

Critical to TBI, whether talking about oscillations at the synapse level (LTP) or through coordinating distal neural activity (phase coherence), theta disruption impairs plasticity and learning. When neural activity in the medial septum is depressed pharmacologically, rats perform poorly on the radial arm [201], T [202], and Morris water mazes [203]. Low theta phase coherence is related to cognitive dysfunction in rats [204,205] and humans [206]. Phase precession is disrupted in aging rats [207,208] and in epileptic rats [209]. Hippocampal interneurons involved in theta oscillations are vulnerable to cell death after TBI [210,211,212]. There is also evidence that TBI alone can change activity in inter- and CA1 hippocampal neurons [213,214,215]. We found that theta power and theta coherence are disrupted for days to weeks after mild [85] or moderate LFP [144,216] inductions of TBI. Importantly, stimulating the medial septum to strengthen *theta* oscillations improved cognitive performance in rats with TBI [145,146] and epilepsy [142,143]. Thus, theta oscillations are important for cognition, but TBI disrupts them, and strengthening theta improves cognition. Therefore, disrupted theta oscillations are a potential mechanism that can explain changes in cognitive function following mTBI. Interventions that restore theta oscillations, power, coherence, and phase–amplitude coupling may effectively treat patients with mTBI, even months to years post-injury.

## 13. Theta as a Potential Target of Neurostimulation in Neurotypical Humans

Neuromodulatory approaches, most commonly tDCS, can improve task performance. TDCS involves the application of an electrical current through two scalp electrodes with current flowing between them [126,217]. The strength and breadth of the current field can be modified by modeling current flow [218] and tailoring current appropriately for pathological conditions [219] and to reach deeper targets [220,221]. It is appealing for translational use because it is affordable, could be self-administered at home, and has a strong safety profile.

In humans, some evidence shows that neuromodulation alters theta activity. For example, cognitive *training* paired with several neuromodulation approaches (e.g., tDCS, tACS) is beneficial to healthy and clinical populations [126,217,222,223,224,225,226,227]. This combined approach can elicit task transfer [228,229], the most highly sought outcome.

To identify the underlying mechanism of improvement, it is helpful to pair neuroimaging with neuromodulation. Combined EEG and tDCS studies reveal that improved WM is due to enhanced theta attributes, including phase synchrony, phase–amplitude coupling, and theta-gamma cross-frequency coupling [230,231,232]. Transcranial alternating current (tACS) *also* strengthens theta synchrony [233] but requires frequency-specific tailoring to the individual [234]. Other forms of neuromodulation, including transcranial random noise stimulation (tRNS) and vagus nerve stimulation (VNS), are beginning to be used to address the physical and psychiatric symptoms of TBI [235]. It is worth noting that there are concerns regarding neuromodulation-induced changes that might *harm* cognition [236] because the current flow is diffuse and, therefore, can influence many regions. Determining how neuromodulation can realign theta and other neural patterns holds promise in TBI. Importantly, there are other possible mechanisms, as there are many effects of neurostimulation on the brain. For instance, changes in other frequency bands, such as reduced delta activity, after tDCS in moderate TBI patients were associated with superior cognitive outcomes [237]. More work is needed to identify the relevant measurements associated with behavioral improvement.

## 14. Cautionary Tale: Getting Ahead of the Data

The sheer prevalence of mTBI/rmTBI and encouraging findings from non-invasive brain stimulation approaches put pressure on bench-to-beside translation. Currently, in the United States, several TMS devices and protocols are federally approved to treat treatment-resistant depression, obsessive compulsive disorder, anxious depression, anxiety, and migraine. Beyond these approved protocols, a growing hoard of fee-for-service clinics offers off-label rTMS services for conditions as diverse as pediatric mental health and autism, insomnia, dementia, chronic pain, tinnitus, dementia, and stroke recovery. There is also a vibrant do-it-yourself brain-hacking community providing online instructions for building and applying your own device. Individual clinicians are moving more quickly than the research consensus or federal regulatory agencies, but the collective view remains cautiously optimistic that neurostimulation techniques will bear fruit [238].

In the field of TBI, there are clear opportunities as well as threats pertaining to the translation of neurostimulation techniques, whether *non*-invasive rTMS and tDCS or invasive DBS. For example, there was a potentially groundbreaking case study where central thalamic DBS rescued a patient from a persistent minimally conscious state [239]. However, in a subsequent study of 14 patients, DBS did not improve outcomes [240]. Perhaps what differentiated the two studies was neuroanatomy. In the patients with improved outcomes, their neuroanatomy was largely intact following injury, whereas patients with larger lesions did not improve. It is critical to consider which circuits are damaged by TBI and to what extent. Are there remaining connections or cortical regions available to stimulate non-invasively? Or is the only viable target subcortical, such as the medial septum or nucleus accumbens? Can intermittent stimulation lead to persistent benefits, or is continuous stimulation required to maintain a cognitive benefit? Invasive neurosurgery for someone with a subtle but significant mTBI-related attention disorder may not make sense, whereas non-invasive stimulation may significantly improve outcomes. Patients with more severe dysfunction may require continuous stimulation, similar to patients with Parkinson’s disease or epilepsy, and therefore might respond better to an implantable stimulator, regardless of whether the stimulation is from an extradural or depth electrode. If we, as scientists and clinicians, take a haphazard approach and ignore details such as the target behavior and the severity of the underlying injury, we risk rejecting technologies that may benefit a subset of patients or advance a study based on a case study into a patient population who will be unlikely to benefit.

Another opportunity involves researching and optimizing stimulation parameters for those with mood disorders experiencing cognitive deficits due to mTBI and an adjacent study testing patients with moderate/severe TBI, e.g., [241,242,243,244,245]. Although the focus of this review was solely on cognition, hmTBI is highly comorbid with anxiety or depressive disorders, especially within the Veteran community [246]. Unfortunately, results from rTMS studies are mixed, with some showing some evidence of benefit on some measures [243] and others showing little or no improvement [247]. One possible explanation could be differences in protocols. Whereas these studies stimulated over the left dorsolateral prefrontal cortex, they used a different number of pulses and pulse amplitudes. A highly researched topic following mTBI is neuromodulation to alleviate post-concussive headache, and again, varying stimulation techniques have been deployed in these studies [101,248,249,250]. Because mTBI is heterogeneous, the addition of a mood disorder or other condition creates a difficult empirical question, as intervention technique [113,251] and timing [252] can be important contributing factors to experimental outcomes.

## 15. Conclusions

Our goal in reviewing TBI response to neurostimulation was to highlight limitations attributable to heterogeneous approaches and to raise the possibility of a more efficient, efficacious path. Similarly, previous reports have noted a wide variety of stimulation parameters have hindered the ability to draw comparisons between protocols and outcomes [1]. To accelerate the research and federal consensus on the mechanisms and proper research parameters for each condition, more interdisciplinary research that is mechanistically focused is needed. Specifically, this mechanical focus should systematically assess treatment parameters to optimize the rehabilitation potential for those experiencing cognitive deficits following an mTBI. Such collaborative projects between basic and clinical science will facilitate crossing scales and uncovering patterns that can improve clinical care decisions for clinicians, parents, coaches, and the broader community. While human trials in both cognition and neuroscience can provide a knowledge base on clinical efficacy, basic science in rodents is needed to create a deeper understanding of the *mechanism* to achieve efficacious treatment parameters.

As an example, we described how, in both humans and rats, theta oscillations are critical to plasticity and learning and that attenuated theta (i.e., power, synchrony) correlates with impaired function. Therefore, aligning, restoring, or strengthening theta rhythms shows promise in rodents and humans, and we should follow up on these successes with renewed vigor. Moreover, it is critical to integrate translational tools, such as EEG, MRI, or PET, that can cross scales from rodents to large animal models to humans. Not only are these tools diagnostic, perhaps helping to identify those patients who would most benefit from therapy, but they are also theragnostic, as only EEG, for example, can determine whether modulation has influenced oscillatory activity. Despite the innate difficulties of collaborative, interdisciplinary work and the known difficulty in predicting human response based on rodent findings [253], more research is needed in this effort. After two decades of exploratory research using neurostimulation, a stronger theoretical and mechanistic framework is needed to alleviate the consequences of TBI.
